# Numerous Items

**Published:** 1896-08

**Authors:** 


### Medical News and Miscellany.

**College of Physicians of Philadelphia.**—The William F. Jenks prize, the fourth triennial prize, of four hundred dollars, under the deed of trust of Mrs. William F. Jenks, will be awarded to the author of the best essay on "The etiology and pathology of diseases of the endometrium, including the septic inflammations of the puerperium."

The conditions annexed by the founder of this prize are, that the "prize or award must always be for some subject connected with obstetrics, or the diseases of women, or the diseases of children," and that "the trustees, under this deed for the time being, can, in their discretion, publish the successful essay, or any paper written upon any subject for which they may offer a reward, provided the income in their hands may, in their judgment, be sufficient for that purpose, and the essay or paper be considered by them worthy of publication. If published, the distribution of said essay shall be entirely under the control of said trustees. In case they do not publish the said essay or paper, it shall be the property of the College of Physicians of Philadelphia."

The prize is open for competition to the whole world, but the essay must be the production of a single person.

The essay, which must be written in the English language, or if in a foreign language, accompanied by an English translation, must be sent to the College of Physicians of Philadelphia, Pennsylvania, U. S. A., before January 1, 1898, addressed to Barton Cooke Hirst, M. D., Chairman of the William F. Jenks Prize Committee.

Each essay must be typewritten, distinguished by a motto, and accompanied by a sealed envelope bearing the same motto, and containing the name and address of the writer. No envelope will be opened except that which accompanies the successful essay.

The committee will return the unsuccessful essays if reclaimed by their respective writers, or their agents, within one year.

The committee reserves the right not to make an award if no essay submitted is considered worthy of the prize.

JAMES V. INGRAM,
May 1, 1896. Secretary of the Trustees.

---

**Couldn't Stand the Pressure.**—The Equitable Life Assurance Society has announced to all its medical examiners that from and after July 1 (ult.), they will pay "the uniform fee of $5 for each completed examination report and opinion of risk." Thought so.

---

**A Lusus Naturæ.**—A well authenticated case is going the rounds of the press of a dermoid cyst in the abdomen of a young man, which, upon being removed by operation, was found to contain a five-months female fetus, supposed to have been a twin sister. The young man did not survive the operation, and never knew the nature of his tumor. A brief account is given in the *New York Medical Record* of the wonderful case—issue of July 11, 1896.

---

**The** Secretary, in the announcement of the Pan-American Medical Congress to be held in Mexico in November, says, "the session will be a solemn one." We suppose it will be a memorial service over the dead, as preliminary to business.

---

**Resigned.**—As we close our last form, it is announced that Dr. C. T. Simpson, superintendent of the State lunatic asylum at Austin, has resigned. It is understood that he has purchased an interest in Dr. Threadgill's private asylum in the Indian Territory, where Dr. Threadgill has some kind of contract with the United States government for taking care of insane Indians. A successor has not been appointed up to the time the JOURNAL goes to press, July 27th. Indications point to Dr. B. M. Worsham, the former first assistant at that institution, and now superintendent of the Southwestern Insane Asylum, which will now create a vacancy at the San Antonio asylum. It will be filled, probably, by some physician from the southern or southwestern portion of the State.

---

**Von Boeckmann, the Journal's** printer, has been awarded the contract for the Texas State Medical Association Vol. of Transactions for 1896, on the lowest and best bid over numerous competitors, his bid for 550 copies in paper (estimate 350 pages), being $363, or about 66 cents per copy; he to bind ten presentation copies in handsome morocco and gilt free. Last year's volume of 350 pages, 500 copies in cloth, cost about $575, exclusive of postage and wrapping, or about $1.25 per copy. This results in a saving of $225 on the printing, nearly 40 per cent. It will be remembered that prior to Secretary West's incumbency Von Boeckmann had for several years gotten out the Transactions, and his work was always satisfactory and his prices far below those paid afterwards—he producing a handsome cloth bound volume of 400 pages, for about 80 cents.

The JOURNAL may be taken as a fair specimen of his work. In the last year his facilities for book making have been greatly improved. He has added a third story to his large brick building and fitted it up as a model bindery; has put in new power presses and labor saving devices, all of the latest kind, and on the ground floor he carries an immense stock of book-findings, blank books, paper, stationery, etc. There is no more extensive or more complete establishment in the South than this. All his type-setting is done by hand, and his establishment is non-union. He keeps his hands at work all through the dead season, when others discharge them; and this is one reason why he can do such fine work at such low rates. The Association is to be congratulated.

---

**A Rare Opportunity** is offered a competent and energetic physician to secure one of the best locations to practice to be found anywhere. Dr. Sam R. Burroughs, who has held the practice of the larger part of east Leon county for twenty-five years, has been induced to go to the railroad (Buffalo) for convenience of patients, and offers, for less than cost of residence, his home, consisting of a well improved farm of 206 acres, with growing crops, seven-room residence nearly new, out houses, bath house, milk house, stables, three tenant houses, fine orchard, his office and equipments—all for $1500. His practice and good will go to the purchaser. Churches and schools; healthy home beautifully improved. Here is a rare chance to secure a lovely home and paying practice for a small consideration. Address Dr. Burroughs, at Buffalo, Leon county, Texas, or this journal.

**Von Boeckmann** authorizes us to say that the forthcoming Transactions being in paper, he will bind it, if desired, on individual orders for 50 cents per volume. Every member is entitled to a copy, and if orders are sent in before the work is delivered to the Secretary it will save express charges both ways.

